# The frequency and clinical impact of HER2 alterations in lung adenocarcinoma

**DOI:** 10.1371/journal.pone.0171280

**Published:** 2017-02-01

**Authors:** Eun Kyung Kim, Kyung A. Kim, Chang Young Lee, Hyo Sup Shim

**Affiliations:** 1 Department of Pathology, Severance Hospital, Yonsei University College of Medicine, Seoul, Korea; 2 Department of Cardiovascular and Thoracic Surgery, Severance Hospital, Yonsei University College of Medicine, Seoul, Korea; Institut national de la recherche scientifique, CANADA

## Abstract

Human epidermal growth factor receptor 2 (HER2 or ErbB2) can be overexpressed, amplified and/or mutated in malignant tumors, and is a candidate for therapeutic targeting. However, molecular associations and clinical significances of these alterations were controversial in lung cancer. In this study, we investigated the frequency and clinicopathological significance of HER2 dysregulation in patients with lung adenocarcinoma. HER2 protein overexpression, gene amplification, and gene mutation were evaluated by immunohistochemistry (IHC), silver in situ hybridization, and direct sequencing, respectively. The H-scoring method and American Society of Clinical Oncology/College of American Pathologists breast cancer guidelines were used to interpret IHC results. Genetic analyses of *EGFR* and *KRAS* mutations, and of *ALK* and *ROS1* rearrangements, were also performed. Of the 321 adenocarcinoma patients identified, HER2 overexpression (H-score ≥200) and gene amplification were found in 6 (1.9%) and 46 (14.3%), respectively. HER2 overexpression was correlated with papillary predominant histology; furthermore, it indicated poor overall survival and was an independent prognostic factor. *HER2* amplification was associated with pleural invasion and showed a tendency towards shorter overall and disease-free survival. High-level gene amplification (HER2/CEP17 ratio ≥5 or copy number ≥10) was a poor prognostic factor for disease-free survival. *HER2* mutations were detected in 6.7% (7 of 104) of driver oncogene-negative adenocarcinomas. Our study suggests that HER2 overexpression or amplification is a poor prognostic factor in lung adenocarcinoma, although the frequency of such events is low. Since molecular targeted agents are being tested in clinical trials, awareness of the specific HER2 status can influence the prognostic stratification and treatment of patients with molecularly defined subsets of lung adenocarcinoma.

## Background

Lung cancer is estimated to be responsible for more than one-quarter (27%) of all cancer-related deaths worldwide [1]. Molecular-based research and systematic genomic studies of this disease have revealed several driver mutations such as those of the epidermal growth factor receptor (*EGFR*) as well as rearrangements of the anaplastic lymphoma kinase (*ALK*) gene; tyrosine kinase inhibitors (TKIs) have been developed against the proteins encoded by these genes. More recently, comprehensive molecular profiling of lung adenocarcinoma from The Cancer Genome Atlas data has identified additional driver gene alterations, including amplifications in human epidermal growth factor receptor 2 (*HER2* or *ERBB2*) [2].

HER2 is a receptor tyrosine kinase and a member of the human EGFR (ErbB) family. It is encoded by the *HER2* gene located on the long arm of chromosome 17 (17q21), and activates downstream signaling pathways such as those involving PI3K-Akt and MEK/ERK to elicit cell proliferation and migration [3]. Many breast and gastric cancers have been found to carry *HER2* amplifications, and the protein is overexpressed in these tumors. Monoclonal antibodies directed against HER2, such as trastuzumab (Herceptin), has improved patient outcomes [4,5].

*HER2* genetic alterations have also been described in non-small cell lung cancer (NSCLC). Gene amplification is found in 10–20% of these cancers, while HER2 protein overexpression has been observed in 2.4–38% [6–11]. Moreover, *HER2* mutations, such as in-frame insertions, have been detected in 2–4% of lung adenocarcinomas [2,12,13]. However, the molecular associations of *HER2* gene amplification, mutation, and HER2 protein overexpression in lung cancers were controversial [10,14,15]. Although clinical trials of HER2-targeting agents have produced disappointing results, certain subgroups of patients with high HER2 expression, gene amplification, or mutations have shown good responses to HER2-targeted therapy [16–20]. Additional novel drugs are also under ongoing investigation.

In this study, we aimed to investigate clinicopathological characteristics and implications of HER2 protein overexpression and gene amplification in NSCLC. Additionally, we performed mutational analysis of *HER2* in a subset of adenocarcinoma, and examined correlations with other genetic alterations.

## Materials and methods

### Patients and clinical samples

Archived formalin-fixed, paraffin-embedded (FFPE) primary tumor tissues were obtained from consecutive NSCLC patients who underwent surgical resection at our institution between 2005 and 2011. Patients who had undergone preoperative treatment or had another malignancy within the 5 years prior to NSCLC diagnosis, or else had inadequate tissue samples or insufficient clinical data, were excluded. Clinical data were collected and reviewed from the patient records. Histologic features were evaluated by two pathologists (H.S.S and E.K.K.) and classified according to the Seventh American Joint Committee on Cancer TNM cancer classification system [21] and the World Health Organization 2015 criteria [22]. The median follow-up period was 62 months (range: 1–126 months) after surgical resection. This retrospective study was approved by the Institutional Review Board of Severance Hospital (No. 4-2015-0561).

### Tissue microarray preparation

Sections of FFPE tissues were prepared and stained with hematoxylin and eosin. Areas representative of the tumor were selected and sampled to construct tissue microarrays (TMAs) under a microscope. Two different cores per case (2–3 mm in diameter) were punched out from donor blocks and placed into 6×5 or 8×6 recipient blocks. The core areas of all TMA blocks were confirmed to contain at least 25% tumor tissue.

### Immunohistochemistry and scoring criteria

Protein expression was evaluated using the PATHWAY anti-HER2/neu (4B5) rabbit monoclonal primary antibody (Ventana Medical Systems, Tucson, AZ, USA). Immunohistochemistry (IHC) was performed on 4-μm TMA tissue sections by using the Ventana Bench Mark XT Autostainer (Ventana Medical Systems, Tucson, AZ, USA). HER2 IHC was assessed using two different methods: the H-scoring system [23] and American Society of Clinical Oncology/College of American Pathologists (ASCO/CAP) guidelines of breast cancer [24]. Semiquantitative H-scoring assessment is performed by multiplying staining intensity (0, none; 1, weak or barely detectable; 2, moderate; and 3, strong) with the percentage of positive cells (0–100%). H-scoring took into account both membranous and cytoplasmic staining. The final scores ranged from 0 to 300 and were classified as high (200 ≤ H-score ≤ 300), intermediate (100 ≤ H-score < 200), low (0 < H-score < 100), and negative (H-score = 0). As for the ASCO/CAP breast cancer guidelines, the final score ranged between 0 and 3 based on membranous staining (0: no staining or incomplete membrane staining that is faint/barely perceptible and comprises ≤10% of the invasive tumor cells; 1+: incomplete membrane staining that is faint/barely perceptible and comprises >10% of the invasive tumor cells; 2+: circumferential membrane staining that is incomplete and/or weak/moderate and encompasses >10% of the invasive tumor cells, or complete and circumferential membrane staining that is intense but comprises ≤10% of the invasive tumor cells; and 3+: circumferential membrane staining that is complete and intense). Scores of 0 and 1 were considered negative; scores of 2 and 3 were considered equivocal and positive, respectively.

### Silver in situ hybridization and interpretation

Silver in situ hybridization (SISH) was performed using the INFORM HER2 Dual ISH DNA Probe Cocktail assay (Ventana Medical Systems) with an automated slide stainer according to the manufacturer’s protocols (BenchMark XT; Ventana Medical Systems). Black (HER2) and red (CEP17) signals were quantified in 60 non-overlapping tumor cells. According to the ASCO/CAP guidelines for dual-probe ISH [24], interpretation of *HER2* gene amplification was defined as follows: negative, *HER2*/*CEP17* ratio <2.0 with an average *HER2* copy number <4.0 signals/cell; equivocal, *HER2*/*CEP17* ratio <2.0 with an average *HER2* copy number ≥4.0 but <6.0 signals/cell; positive, *HER2*/*CEP17* ratio ≥2.0 with an average *HER2* copy number ≥4.0 signals per cell or a *HER2*/*CEP17* ratio ≥2.0 with an average *HER2* copy number <4.0 signals/cell or a *HER2*/*CEP17* ratio <2.0 with an average *HER2* copy number ≥6.0 signals/cell. Additionally, tumors harboring an average copy number of ≥10.0 signals/cell or a *HER2*/*CEP17* ratio ≥5.0 were considered to have high-level amplification.

### *HER2*, *EGFR*, and *KRAS* mutation analysis

To determine the *HER2*, *EGFR*, and *KRAS* mutation status, DNA was extracted using the DNeasy isolation kit (Qiagen, Valencia, CA, USA) from FFPE tissues according to the manufacturer’s instructions. For the *HER2* gene, direct DNA sequencing of exons 18 through 21 was performed in a subset of our cohort (n = 60, comprising cases with driver mutation-negative adenocarcinomas). For *EGFR* gene analysis, direct DNA sequencing of exons 18 through 21, or utilizing either the PNAClamp^™^ EGFR Mutation Detection Kit (PANAGENE, Daejeon, Korea) or the PANAMutyper^™^ R EGFR kit (PANAGENE, Daejeon, Korea), was performed in all cases. For *KRAS* gene analysis, direct DNA sequencing of codons 12 and 13, or utilizing the PANAMutyper^™^ R KRAS kit (PANAGENE, Daejeon, Korea) was performed in all cases.

### *ALK* and *ROS1* rearrangement analysis

To identify *ALK* and *ROS1* rearrangements, IHC was performed using the ALK (rabbit monoclonal, clone D5F3, Cell Signaling Technology, Danvers, MA, USA) and ROS1 (rabbit monoclonal, clone D4D6, Cell Signaling Technology, Danvers, MA, USA) antibodies. In IHC positive cases, fluorescence in situ hybridization (FISH) was performed using a break-apart ALK probe (Vysis LSI Dual Color, Break Apart Rearrangement Probe kit, Abbott Molecular, Abbott Park, IL, USA) or ROS1 probe (Abbott Molecular). *ALK* or *ROS1* rearrangements were scored as positive when more than 15% of tumor cells displayed split signals or isolated signals containing a kinase domain (red for ALK and green for ROS1).

### Statistical analysis

The chi-square or Fisher’s exact test was used to evaluate the association between HER2 status and clinicopathological parameters. Correlations between IHC and SISH were estimated using Spearman's correlation coefficients (kappa); kappa values >0.75 denote excellent agreement, values between 0.4 and 0.75 denote fairly good agreement, and values <0.4 denote poor agreement. Overall survival (OS) was assessed from the date of surgery to that of death or the last follow-up visit. Disease-free survival (DFS) was measured from the date of surgery to that of cancer recurrence or the last follow-up visit. Survival rates were calculated using the Kaplan—Meier method, and the differences were analyzed using the log-rank test. Multivariate analysis was performed using the Cox proportional hazards model, which was expressed by the hazard ratio with a 95% confidence interval. P-values <0.05 were considered statistically significant. Statistical analyses were performed using the IBM SPSS 22 software for Windows (IBM Corp, New York, USA).

## Results

### Patient characteristics

According to criteria described above, 419 patients were ultimately selected for analysis: 321 had adenocarcinomas and 96 had squamous cell carcinomas. Characteristics of patients with adenocarcinoma are summarized in Table 1. Lung adenocarcinoma samples were derived from 163 men and 158 women aged 33–90 years (mean = 61.8 years). This population included 196 non-smokers (61.6%) and 125 former or current smokers (38.9%).

**Table 1:** Patient characteristics according to HER2 protein overexpression and gene amplification.

Characteristics, n (%)	Total	Protein overexpression ^a^			Gene amplification ^b^		
		*Positive*	*Negative*	*P value*	*Positive*	*Negative*	*P value*
	321 (100)	6 (1.9)	315 (98.1)		46 (14.3)	275 (85.7)	
Age (years)				0.424			1.000
<62	171 (53.3)	2 (33.3)	169 (53.7)		25 (54.3)	147 (53.5)	
≥62	150 (46.7)	4 (66.7)	146 (46.3)		21 (45.7)	128 (46.5)	
Gender				1.000			0.429
Male	163 (50.8)	3 (50.0)	160 (50.8)		26 (56.5)	137 (49.8)	
Female	158 (49.2)	3 (50.0)	155 (49.2)		20 (43.5)	138 (50.2)	
Smoking status				0.681			0.746
Never	196 (61.1)	3 (50.0)	193 (61.3)		27 (58.7)	169 (61.5)	
Former/current	125 (38.9)	3 (50.0)	122 (38.7)		19 (41.3)	106 (38.5)	
Tumor size (cm)				0.697			0.628
≤3	187 (58.3)	3 (50.0)	184 (58.4)		25 (54.3)	163 (59.3)	
>3	134 (41.7)	3 (50.0)	131 (41.6)		21 (45.7)	112 (40.7)	
Nodal metastasis				0.187			0.242
Positive	111 (34.6)	4 (66.7)	107 (34.0)		20 (43.5)	92 (33.5)	
Negative	210 (65.4)	2 (33.3)	208 (66.0)		26 (56.5)	183 (66.5)	
Pathologic stage				0.104			0.661
I	177 (55.1)	2 (33.3)	175 (55.6)		25 (54.3)	151 (54.9)	
II	58 (18.1)	0 (0)	58 (18.4)		6 (13)	52 (18.9)	
III	79 (24.6)	4 (66.7)	75 (23.8)		14 (30.4)	66 (24)	
IV	7 (2.2)	0 (0)	7 (2.2)		1 (2.2)	6 (2.2)	
Histologic subtype				0.718			0.082
Lepidic	24 (7.5)	0 (0)	24 (7.6)		2 (4.3)	22 (8)	
Acinar	154 (48)	2 (33.3)	152 (48.3)		27 (58.7)	126 (45.8)	
Papillary	41 (12.8)	3 (50.0)	38 (12.1)		7 (15.2)	34 (12.4)	
Micropapillary	30 (9.3)	0 (0)	30 (9.5)		6 (13)	24 (8.7)	
Solid	57 (17.8)	0 (0)	57 (18.1)		4 (8.7)	54 (19.6)	
Mucinous	15 (4.7)	1 (16.7)	14 (4.4)		0 (0)	15 (5.5)	
Papillary predominant type				**0.029**			0.633
Positive	41 (12.8)	3 (50.0)	38 (12.1)		7 (15.2)	34 (12.4)	
Negative	280 (87.2)	3 (50.0)	277 (87.9)		39 (84.8)	241 (87.6)	
Pleural invasion				0.218			**0.035**
Positive	127 (39.6)	4 (66.7)	123 (39.0)		25 (54.3)	103 (37.5)	
Negative	194 (60.4)	2 (33.3)	192 (61.0)		21 (45.7)	172 (62.5)	
*EGFR* mutation				0.215			0.635
Positive	157 (48.9)	1 (16.7)	156 (49.5)		24 (52.2)	132 (48)	
Negative	164 (51.1)	5 (83.3)	159 (50.5)		22 (47.8)	143 (52)	
*KRAS* mutation				0.112			0.595
Positive	32 (10)	2 (33.3)	30 (9.5)		3 (6.5)	29 (10.5)	
Negative	289 (90)	4 (66.7)	285 (90.5)		43 (93.5)	246 (89.5)	
*ALK* rearrangement				1.000			0.329
Positive	20 (6.2)	0 (0)	20 (6.3)		1 (2.2)	19 (6.9)	
Negative	301 (93.8)	6 (100)	295 (93.7)		45 (97.8)	256 (93.1)	
*ROS1* rearrangement				1.000			0.607
Positive	8 (2.5)	0 (0)	8 (2.5)		0 (0)	8 (2.9)	
Negative	313 (97.5)	6 (100)	307 (97.5)		46 (100)	267 (97.1)	

^a^ HER2 protein overexpression denotes H-score≥200.

^b^ *HER2* gene amplification denotes *HER2/CEP17* ratio≥2.0 or average *HER2* copy number≥6.0 signals/cell.

### HER2 overexpression and its clinicopathological significance

**Fig 1:**
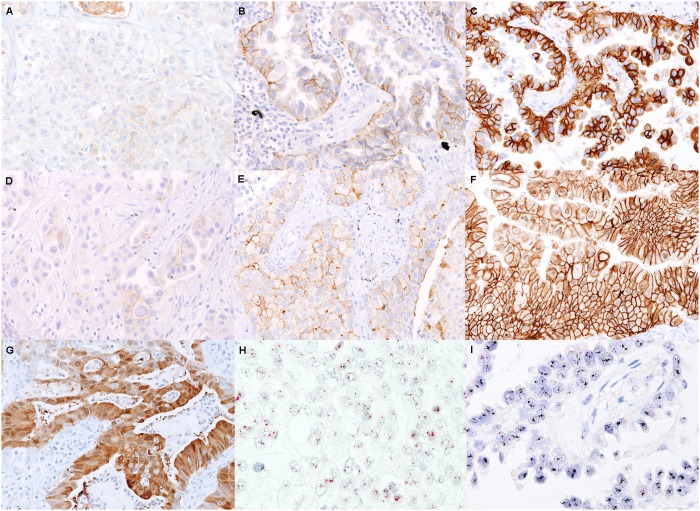
Representative images of HER2 immunohistochemistry and *HER2* silver in situ hybridization. Shown are negative/low (A), intermediate (B), and high (C) HER2 expression levels according to the H-scoring system; and HER2 expression scores of 0/1+ (D), 2+ (E), and 3+ (F) according to the ASCO/CAP breast cancer guidelines. A case with predominantly cytoplasmic staining is shown in (G). Silver in situ hybridization with no amplification (H; which is the same case as ‘A’) and high-level amplification (I; which is the same case as ‘C’).

Of 321 adenocarcinomas, membranous or cytoplasmic HER2 expression was classified as negative in 192 cases (59.8%), low in 98 (30.5%), intermediate in 25 (7.8%), and high in 6 (1.9%) by the H-scoring method (Fig 1). Membranous expression of HER2 was scored as 0 in 248 samples (77.3%), 1+ in 48 (15.0%), 2+ in 21 (6.5%), and 3+ in 4 (1.2%) according to the ASCO/CAP guidelines of breast cancer. Cytoplasmic expression without membranous staining was observed in 26 adenocarcinomas (8.1%). Among them, only one case showed a high H-score, but did not exhibit *HER2* amplification.

Clinicopathological findings observed in lung adenocarcinomas with or without HER2 overexpression are summarized in Table 1. High expression of HER2 (H-score ≥200) was more frequently found in adenocarcinomas with a predominantly papillary histology (P = 0.029). Among HER2-overexpressing adenocarcinomas, 1 (16.7%) and 2 (33.3%) had *EGFR* and *KRAS* mutations, respectively, while none had *ALK* or *ROS1* rearrangements.

### *HER2* gene amplification and its clinicopathological significance

Of 321 adenocarcinomas, the results of SISH were negative in 271 (84.4%), equivocal in 4 (1.2%), and positive in 46 (14.3%) according to the criteria described above (Fig 1). Clinicopathological findings observed in lung adenocarcinomas with or without *HER2* gene amplification are summarized in Table 1. Adenocarcinomas with pleural invasion were significantly correlated with *HER2* amplification (P = 0.035). Among *HER2*-amplified adenocarcinomas, 24 (52.2%) and 3 (6.5%) had *EGFR* and *KRAS* mutations, respectively, while 1 (2.2%) showed *ALK* rearrangement. Five cases (1.6%) were found to have high-level amplification. While the statistical power was low owing to the small number of cases with high-level amplification, these cases tended to correlate with large tumor sizes (P = 0.078), pleural invasion (P = 0.065), and lymphovascular invasion (P = 0.053).

### *HER2* mutations

*HER2* mutations were detected in 7 of 104 driver mutation-negative (i.e., *EGFR/KRAS/ALK/ROS1* negative) adenocarcinomas examined (6.7%), and included mostly insertion mutations in exon 20. The mutation types and clinicopathological features were summarized in Table 2. The patients were mostly women or never-smokers. The range of *HER2*/*CEP17* ratios and the *HER2* copy numbers were from 1.4 to 2.4 and from 3.2 to 4.8, respectively. No high-level amplification was found in *HER2* mutated tumors.

**Table 2:** Summary of *HER2* mutated cases.

Case No.	*HER2* mutation type	Age	Gender	Smoking	Histologic subtype	Stage	*HER2* SISH ratio	*HER2* copy number
1	p.A775_G776 insYVMA (c. 2324_2325 ins12)	64	Female	Never	Acinar	IIIa	1.7	3.5
2	p.G776>VC (c.2326_2327 insTGT)	55	Female	Never	Acinar	IIIa	2.2	4.5
3	p.A775_G776 insYVMA (c. 2324_2325 ins12)	80	Female	Never	Acinar	Ia	2.2	4.4
4	p.P780_Y781insGSP (c.2339_2340 ins9)	79	Female	Never	Micropapillary	Ib	1.7	3.5
5	p.A775_G776 insYVMA (c. 2324_2325 ins12)	81	Female	Former	Acinar	IIIa	1.4	3.2
6	p.A775_G776 insYVMA (c. 2324_2325 ins12)	55	Female	Never	Papillary	IIIa	2.2	4.4
7	p.G776S(c. 2326 G>A) & p. V777_G778 insE(c. 2332_2333 insAGG)	58	Male	Never	Acinar	IIIa	2.4	4.8

### Survival analysis of HER2 overexpression and gene amplification in adenocarcinoma

**Fig 2:**
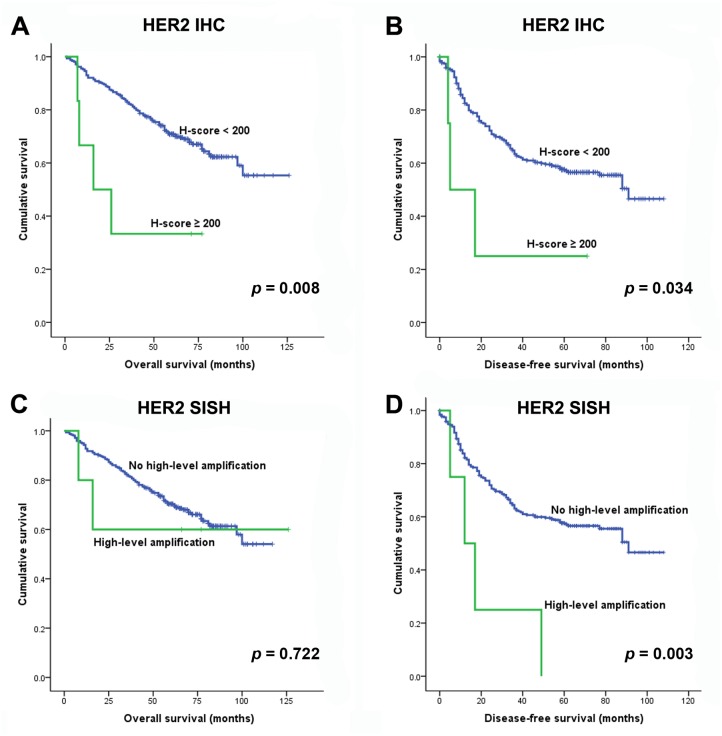
Overall and disease-free survival curves according to the HER2 immunohistochemistry H-score (A and B, respectively) and according to high-level amplification by *HER2* as detected by silver in situ hybridization (C and D, respectively).

Patients with overexpression of HER2 showed significantly shorter OS and DFS rates compared to patients without HER2 overexpression according to the H-scoring method (P = 0.008 and 0.034, respectively; Fig 2). Patients with amplification of the *HER2* gene tended to have shorter (albeit non-significant) OS rates compared to those without amplification (P = 0.233). Patients with ‘high-level’ amplification of the *HER2* gene exhibited shorter DFS rates (P = 0.003; Fig 2).

Univariate analysis revealed that male sex (P<0.001), older age (≥62 years; P = 0.017), larger tumor size (≥3 cm; P<0.001), smoking (P<0.001), a solid predominant type tumor (P = 0.002), pleural invasion (P = 0.003), lymphovascular invasion (P<0.001), lymph node metastasis (P<0.001), advanced pTNM stage (P<0.001), and HER2 protein overexpression (H-score ≥200; P = 0.008) were significantly correlated with poor OS. Shorter DFS rates were related to younger age (<62 years; P = 0.003), larger tumor size (≥3 cm; p<0.001), solid predominant type tumors (P = 0.011), micropapillary predominant type tumors (P = 0.007), pleural invasion (P = 0.001), lymphovascular invasion (P<0.001), lymph node metastasis (P<0.001), advanced pTNM stage (P<0.001), HER2 protein overexpression (H-score ≥200; P = 0.034), and *HER2* high-level amplification (P = 0.003). Multivariate analysis for OS identified male sex, older age, large tumor size, solid predominant type, advanced pTNM stage, and HER2 overexpression as independent prognostic factors (Table 3). Poor DFS was independently associated with younger age, larger tumors, lymph node metastasis, and advanced pTNM stage (Table 3).

**Table 3:** Multivariate survival analyses.

Overall survival			
Variables	P value	HR^a^	95% CI^b^
Male	0.004	1.851	1.224–2.800
Age ≥ 62	0.004	1.832	1.215–2.761
Size ≥ 3cm	0.025	1.602	1.060–2.420
Solid predominant type	0.043	1.608	1.015–2.546
HER2 overexpression^c^	0.009	3.999	1.404–11.394
pTNM stage	<0.001	6.432	2.412–17.151
Disease-free survival			
Variables	P value	HR	95% CI
Age < 62	0.001	1.796	1.270–2.538
Size ≥ 3cm	<0.001	2.242	1.573–3.194
Lymph node metastasis	0.028	2.014	1.078–3.762
pTNM stage	0.021	3.521	1.214–10.218

^a.^ HR, hazard ratio;

^b.^ CI, confidence interval;

^c.^ HER2 protein overexpression denotes H-score≥200

### Correlation of IHC with SISH

HER2 protein expression was significantly associated with gene amplification; however, IHC and SISH showed poor concordance (Table 4). The sensitivities and specificities of IHC for detecting amplification were as follows: 23.9% and 92.7% when the H-score cutoff was ≥100, and 23.9% and 94.9% when the cutoff was ≥2+, respectively.

**Table 4:** Correlation between HER2 immunohistochemistry and silver in situ hybridization.

	Total, n (%)	SISH		*P value*	kappa
		*Amplification*	*No amplification*		
*IHC (H score)*				<0.001	0.087
0	192 (59.8)	18 (39.1)	174 (63.3)		
1~99	98 (30.5)	17 (37.0)	81 (29.5)		
100~199	25 (7.8)	8 (17.4)	17 (6.2)		
200~300	6 (1.9)	3 (6.5)	3 (1.1)		
*IHC (membranous)*^a^				<0.001	0.154
0	248 (77.3)	24 (52.2)	224 (81.5)		
1+	48 (15.0)	11 (23.9)	37 (13.5)		
2+	21 (6.5)	8 (17.4)	13 (4.7)		
3+	4 (1.2)	3 (6.5)	1 (0.4)		
Total	321	46	275		

^a^ ASCO/CAP guidelines of breast cancer

## Discussion

In the present study, we investigated HER2 protein expression and genetic alterations in patients with lung adenocarcinomas and their associations with clinicopathological features and prognosis. We observed that the frequency of HER2 overexpression and gene amplification were 1.9% and 14.3% in adenocarcinomas, respectively, and that protein overexpression and high amplification were associated with poor prognosis. In addition, *HER2* mutation was identified in 6.7% of driver oncogene-negative adenocarcinomas.

Previous studies of HER2 overexpression in NSCLC have defined IHC positivity as only membranous expression [10,25–28]. However, we additionally used the H-scoring method that incorporates both cytoplasmic and membranous staining patterns. In adenocarcinomas, 9.7% and 7.7% of tumors showed intermediate or stronger staining (by the H-scoring method) and 2+ or greater expression (by the ASCO/CAP guidelines), respectively. Although the criteria for positivity varied between studies, our positive staining rates are comparable to those observed in previous studies (2.4–38%) [7,10,11]. The percentage of H-scores ≥200 and ASCO/CAP scores of 3+, indicating high expression, were 1.9% and 1.2%, respectively. In contrast to previous studies that showed a correlation between HER2 overexpression and both lymph node metastasis and higher tumor stage [25,26], we found that 50% of HER2-overexpressed tumors were papillary predominant adenocarcinomas. Moreover, an H-score ≥200 group significantly correlated with shorter OS and DFS rates, and was found to be an independent prognostic factor. This result is consistent with previous meta-analyses that have determined HER2 overexpression to be a poor prognostic factor in adenocarcinomas [11,29].

*HER2* amplification was detected by SISH in 14.3% of adenocarcinomas according to the criteria used in breast cancer. A few previous studies using SISH showed similar rates (1.6–20.4%) and revealed female sex, younger age, and papillary-predominant histology to be predictive clinicopathological parameters [10,25,26]. In our cohort, *HER2*-amplified adenocarcinomas more frequently presented with pleural invasion; this group of patients tended to have shorter OS rates (although the difference was not significant). Several investigations that evaluated *HER2* amplification by FISH or SISH also have failed to show a significant relationship between such amplification and prognosis [11,29] However, in our study, high-level amplification (defined as a *HER2*/*CEP17* ratio ≥5.0 or an average HER2 copy number ≥10.0 signals/cell) correlated significantly with poor DFS.

In the present study, HER2 overexpression or gene amplification was not mutually exclusive to other driver mutations. *EGFR* and *KRAS* mutations were simultaneously found in 16.7% and 33.3% of HER2-overexpressing adenocarcinomas, respectively, as well as in 52.2% and 6.5% of *HER2*-amplified adenocarcinomas, respectively. This suggests that HER2 deregulation may be involved in cancer progression and acquired resistance [30]. To that end, recent studies have reported that *HER2* amplification is one of the mechanisms of acquired resistance to EGFR TKIs [30,31]. However, of 104 driver mutation-negative cases, 11 (10.6%) harbored HER2 protein overexpression, *HER2* high-level amplification, or *HER2* gene mutations. Targeted therapies against HER2 activation can be considered as a therapeutic option in patients with such HER2 alterations who have no driver mutations.

*HER2* mutations were detected in 6.7% (7 of 104) of driver mutation-negative adenocarcinomas. Assuming that driver alterations are mutually exclusive, the frequency of *HER2* mutations was 2.2% (7 of 321) in our cohort. Most reported *HER2* mutations involve a A775_G776insYVMA insertion/duplication in exon 20 [12,32] as was observed in 4 of our cases. High-level gene amplification or protein overexpression was not found in *HER2* mutated tumors.

Diagnostic criteria or specific algorithms for selecting patients for HER2 targeted therapy has not yet been established in lung cancer. In the present study, HER2 IHC and SISH were well correlated as shown in previous studies, but demonstrated poor concordance with each other as well as poor sensitivity; this is contrary to findings observed in breast cancers. This suggests that HER2 dysregulation might be more complex in lung cancers than in breast cancers, and that IHC may not be suitable as a screening tool for cases with gene amplification. Therefore, specific HER2 alterations, such as protein overexpression, gene amplification, or genetic mutations, should be investigated for their correlation with drug responsiveness in clinical trials to determine the most robustly predictive biomarkers.

There are limitations for our study. This was a retrospective study, which means that inherent biases may exist. Since the frequency of each alteration is low, further multi-institutional studies are necessary to confirm the results.

## Conclusion

We observed HER2 overexpression and gene amplification in 1.9% and 14.3% of adenocarcinomas, respectively. *HER2* mutations were identified in 6.7% of driver oncogene-negative adenocarcinomas. HER2 overexpression was correlated with papillary predominant histology, and was an independent poor prognostic factor for OS. Moreover, high-level *HER2* amplification was associated with poor DFS. Since targeted agents are currently being tested in clinical trials, determining the HER2 status can influence the treatment of patients with molecularly defined lung adenocarcinoma, as well as provide prognostic stratification.6.7
